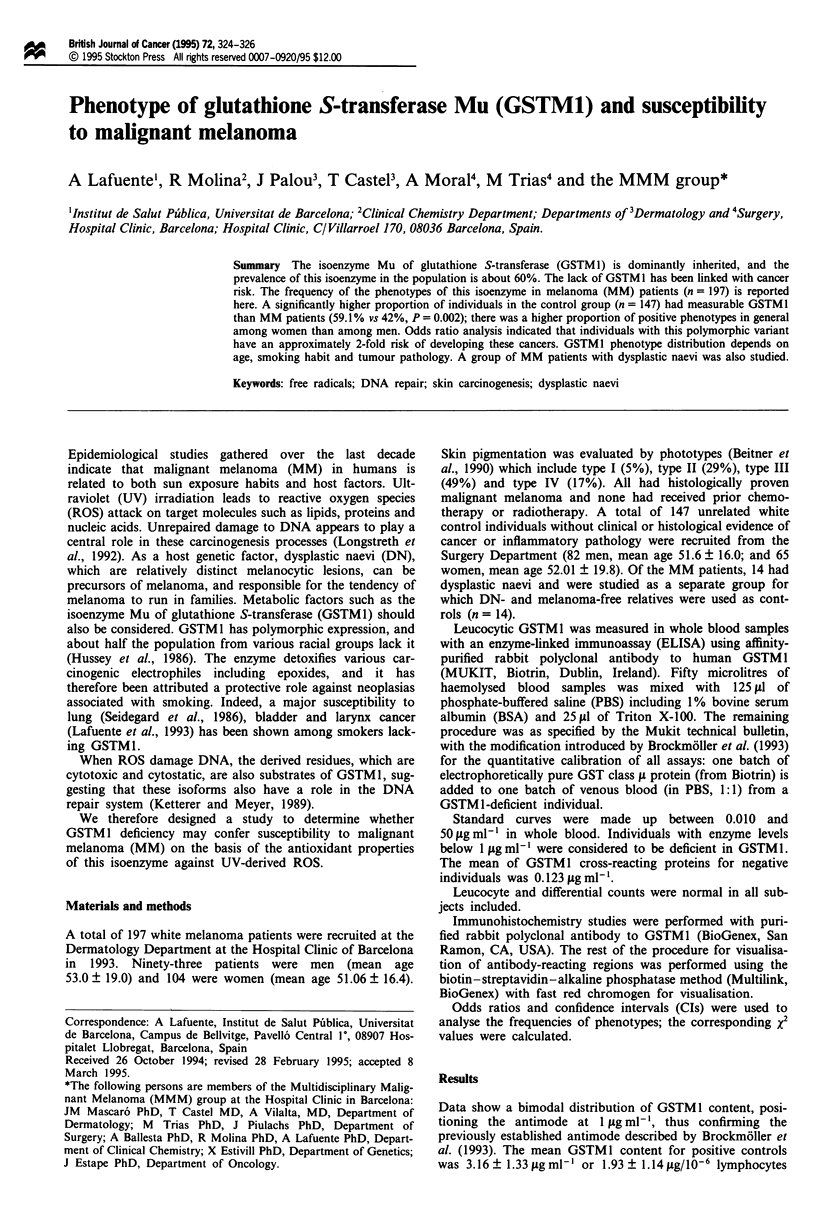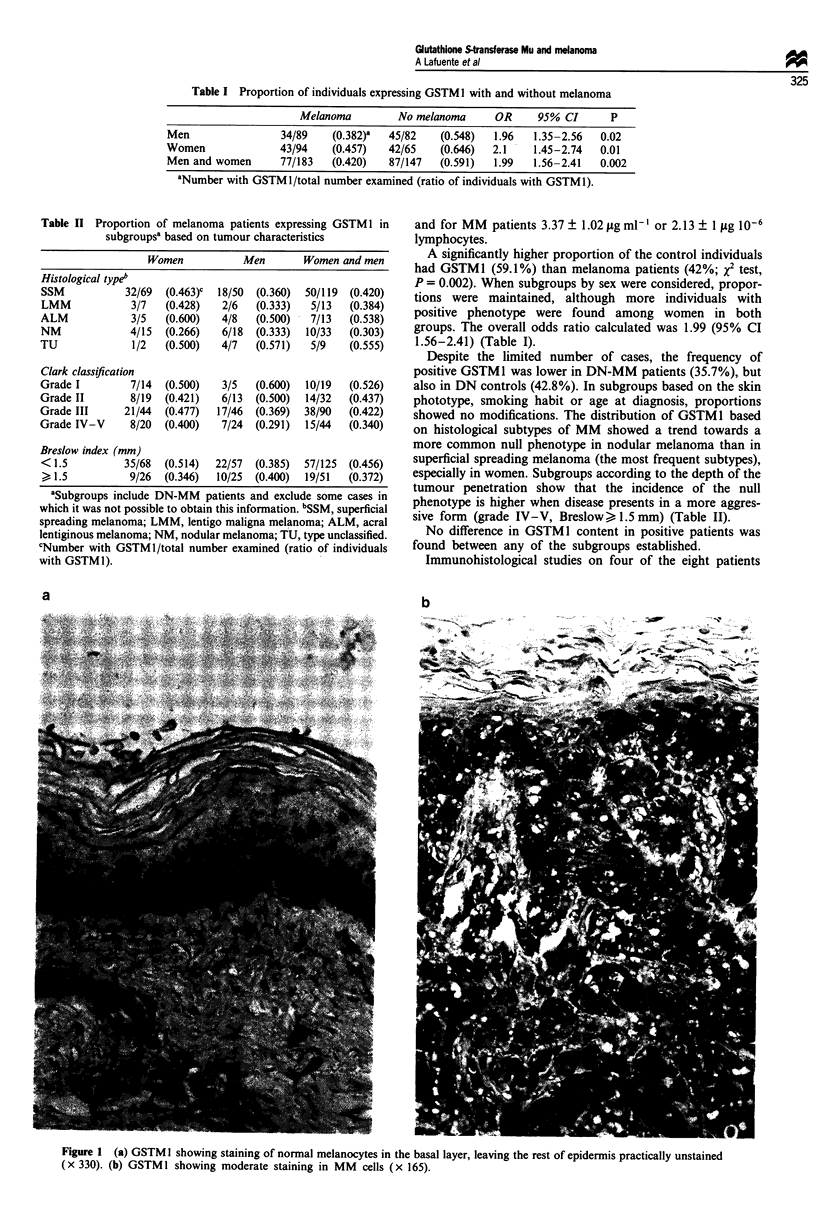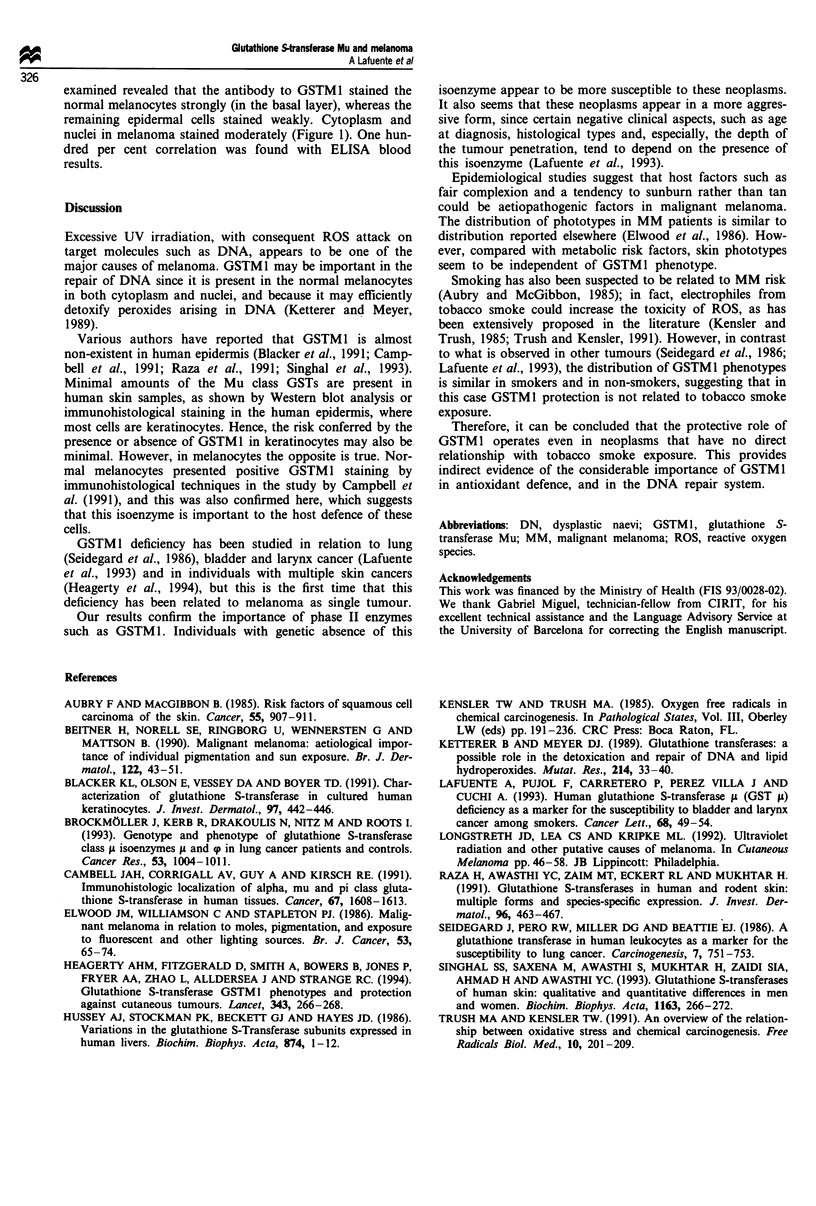# Phenotype of glutathione S-transferase Mu (GSTM1) and susceptibility to malignant melanoma. MMM group. Multidisciplinary Malignant Melanoma Group.

**DOI:** 10.1038/bjc.1995.332

**Published:** 1995-08

**Authors:** A. Lafuente, R. Molina, J. Palou, T. Castel, A. Moral, M. Trias

**Affiliations:** Institute de Salut Pública, Universitat de Barcelona, Spain.

## Abstract

**Images:**


British Journal of Cancer (1995) 72, 324–326
© 1995 Stockton Press All rights reserved 0007-0920/95 $12.00

### Phenotype of glutathione *S*-transferase Mu (GSTM1) and susceptibility to malignant melanoma

A Lafuente[1], R Molina[2], J Palou[3], T Castel[3], A Moral[4], M Trias[4] and the MMM group*

[1]*Institut de Salut Pública, Universitat de Barcelona;* [2]*Clinical Chemistry Department; Departments of* [3]*Dermatology and* [4]*Surgery, Hospital Clinic, Barcelona; Hospital Clinic, C/Villarroel 170, 08036 Barcelona, Spain.*

**Summary** The isoenzyme Mu of glutathione *S*-transferase (GSTM1) is dominantly inherited, and the prevalence of this isoenzyme in the population is about 60%. The lack of GSTM1 has been linked with cancer risk. The frequency of the phenotypes of this isoenzyme in melanoma (MM) patients ($n = 197$) is reported here. A significantly higher proportion of individuals in the control group ($n = 147$) had measurable GSTM1 than MM patients (59.1% *vs* 42%, $P = 0.002$); there was a higher proportion of positive phenotypes in general among women than among men. Odds ratio analysis indicated that individuals with this polymorphic variant have an approximately 2-fold risk of developing these cancers. GSTM1 phenotype distribution depends on age, smoking habit and tumour pathology. A group of MM patients with dysplastic naevi was also studied.

**Keywords**: free radicals; DNA repair; skin carcinogenesis; dysplastic naevi

Epidemiological studies gathered over the last decade indicate that malignant melanoma (MM) in humans is related to both sun exposure habits and host factors. Ultraviolet (UV) irradiation leads to reactive oxygen species (ROS) attack on target molecules such as lipids, proteins and nucleic acids. Unrepaired damage to DNA appears to play a central role in these carcinogenesis processes (Longstreth *et al.*, 1992). As a host genetic factor, dysplastic naevi (DN), which are relatively distinct melanocytic lesions, can be precursors of melanoma, and responsible for the tendency of melanoma to run in families. Metabolic factors such as the isoenzyme Mu of glutathione *S*-transferase (GSTM1) should also be considered. GSTM1 has polymorphic expression, and about half the population from various racial groups lack it (Hussey *et al.*, 1986). The enzyme detoxifies various carcinogenic electrophiles including epoxides, and it has therefore been attributed a protective role against neoplasias associated with smoking. Indeed, a major susceptibility to lung (Seidegard *et al.*, 1986), bladder and larynx cancer (Lafuente *et al.*, 1993) has been shown among smokers lacking GSTM1.

When ROS damage DNA, the derived residues, which are cytotoxic and cytostatic, are also substrates of GSTM1, suggesting that these isoforms also have a role in the DNA repair system (Ketterer and Meyer, 1989).

We therefore designed a study to determine whether GSTM1 deficiency may confer susceptibility to malignant melanoma (MM) on the basis of the antioxidant properties of this isoenzyme against UV-derived ROS.

### Materials and methods

A total of 197 white melanoma patients were recruited at the Dermatology Department at the Hospital Clinic of Barcelona in 1993. Ninety-three patients were men (mean age $53.0 \pm 19.0$) and 104 were women (mean age $51.06 \pm 16.4$). Skin pigmentation was evaluated by phototypes (Beitner *et al.*, 1990) which include type I (5%), type II (29%), type III (49%) and type IV (17%). All had histologically proven malignant melanoma and none had received prior chemotherapy or radiotherapy. A total of 147 unrelated white control individuals without clinical or histological evidence of cancer or inflammatory pathology were recruited from the Surgery Department (82 men, mean age $51.6 \pm 16.0$; and 65 women, mean age $52.01 \pm 19.8$). Of the MM patients, 14 had dysplastic naevi and were studied as a separate group for which DN- and melanoma-free relatives were used as controls ($n = 14$).

Leucocytic GSTM1 was measured in whole blood samples with an enzyme-linked immunoassay (ELISA) using affinity-purified rabbit polyclonal antibody to human GSTM1 (MUKIT, Biotrin, Dublin, Ireland). Fifty microlitres of haemolysed blood samples was mixed with 125 $\mu$l of phosphate-buffered saline (PBS) including 1% bovine serum albumin (BSA) and 25 $\mu$l of Triton X-100. The remaining procedure was as specified by the Mukit technical bulletin, with the modification introduced by Brockmöller *et al.* (1993) for the quantitative calibration of all assays: one batch of electrophoretically pure GST class $\mu$ protein (from Biotrin) is added to one batch of venous blood (in PBS, 1:1) from a GSTM1-deficient individual.

Standard curves were made up between 0.010 and 50 $\mu$g ml$^{-1}$ in whole blood. Individuals with enzyme levels below 1 $\mu$g ml$^{-1}$ were considered to be deficient in GSTM1. The mean of GSTM1 cross-reacting proteins for negative individuals was 0.123 $\mu$g ml$^{-1}$.

Leucocyte and differential counts were normal in all subjects included.

Immunohistochemistry studies were performed with purified rabbit polyclonal antibody to GSTM1 (BioGenex, San Ramon, CA, USA). The rest of the procedure for visualisation of antibody-reacting regions was performed using the biotin–streptavidin–alkaline phosphatase method (Multilink, BioGenex) with fast red chromogen for visualisation.

Odds ratios and confidence intervals (CIs) were used to analyse the frequencies of phenotypes; the corresponding $\chi^2$ values were calculated.

### Results

Data show a bimodal distribution of GSTM1 content, positioning the antimode at 1 $\mu$g ml$^{-1}$, thus confirming the previously established antimode described by Brockmöller *et al.* (1993). The mean GSTM1 content for positive controls was $3.16 \pm 1.33$ $\mu$g ml$^{-1}$ or $1.93 \pm 1.14$ $\mu$g/10$^{-6}$ lymphocytes

Correspondence: A Lafuente, Institut de Salut Pública, Universitat de Barcelona, Campus de Bellvitge, Pavelló Central 1°, 08907 Hospitalet Llobregat, Barcelona, Spain
Received 26 October 1994; revised 28 February 1995; accepted 8 March 1995.

*The following persons are members of the Multidisciplinary Malignant Melanoma (MMM) group at the Hospital Clinic in Barcelona: JM Mascaró PhD, T Castel MD, A Vilalta, MD, Department of Dermatology; M Trias PhD, J Piulachs PhD, Department of Surgery; A Ballesta PhD, R Molina PhD, A Lafuente PhD, Department of Clinical Chemistry; X Estivill PhD, Department of Genetics; J Estape PhD, Department of Oncology.

**Table I** Proportion of individuals expressing GSTM1 with and without melanoma

| | *Melanoma* | | *No melanoma* | | *OR* | *95% CI* | P |
|---|---|---|---|---|---|---|---|
| Men | 34/89 | (0.382)[a] | 45/82 | (0.548) | 1.96 | 1.35–2.56 | 0.02 |
| Women | 43/94 | (0.457) | 42/65 | (0.646) | 2.1 | 1.45–2.74 | 0.01 |
| Men and women | 77/183 | (0.420) | 87/147 | (0.591) | 1.99 | 1.56–2.41 | 0.002 |

[a]Number with GSTM1/total number examined (ratio of individuals with GSTM1).

**Table II** Proportion of melanoma patients expressing GSTM1 in subgroups[a] based on tumour characteristics

| | *Women* | | *Men* | | *Women and men* | |
|---|---|---|---|---|---|---|
| *Histological type*[b] | | | | | | |
| SSM | 32/69 | (0.463)[c] | 18/50 | (0.360) | 50/119 | (0.420) |
| LMM | 3/7 | (0.428) | 2/6 | (0.333) | 5/13 | (0.384) |
| ALM | 3/5 | (0.600) | 4/8 | (0.500) | 7/13 | (0.538) |
| NM | 4/15 | (0.266) | 6/18 | (0.333) | 10/33 | (0.303) |
| TU | 1/2 | (0.500) | 4/7 | (0.571) | 5/9 | (0.555) |
| *Clark classification* | | | | | | |
| Grade I | 7/14 | (0.500) | 3/5 | (0.600) | 10/19 | (0.526) |
| Grade II | 8/19 | (0.421) | 6/13 | (0.500) | 14/32 | (0.437) |
| Grade III | 21/44 | (0.477) | 17/46 | (0.369) | 38/90 | (0.422) |
| Grade IV–V | 8/20 | (0.400) | 7/24 | (0.291) | 15/44 | (0.340) |
| *Breslow index (mm)* | | | | | | |
| $<1.5$ | 35/68 | (0.514) | 22/57 | (0.385) | 57/125 | (0.456) |
| $\geqslant 1.5$ | 9/26 | (0.346) | 10/25 | (0.400) | 19/51 | (0.372) |

[a]Subgroups include DN-MM patients and exclude some cases in which it was not possible to obtain this information. [b]SSM, superficial spreading melanoma; LMM, lentigo maligna melanoma; ALM, acral lentiginous melanoma; NM, nodular melanoma; TU, type unclassified. [c]Number with GSTM1/total number examined (ratio of individuals with GSTM1).

and for MM patients $3.37 \pm 1.02\ \mu g\ ml^{-1}$ or $2.13 \pm 1\ \mu g\ 10^{-6}$ lymphocytes.

A significantly higher proportion of the control individuals had GSTM1 (59.1%) than melanoma patients (42%; $\chi^2$ test, $P = 0.002$). When subgroups by sex were considered, proportions were maintained, although more individuals with positive phenotype were found among women in both groups. The overall odds ratio calculated was 1.99 (95% CI 1.56–2.41) (Table I).

Despite the limited number of cases, the frequency of positive GSTM1 was lower in DN-MM patients (35.7%), but also in DN controls (42.8%). In subgroups based on the skin phototype, smoking habit or age at diagnosis, proportions showed no modifications. The distribution of GSTM1 based on histological subtypes of MM showed a trend towards a more common null phenotype in nodular melanoma than in superficial spreading melanoma (the most frequent subtypes), especially in women. Subgroups according to the depth of the tumour penetration show that the incidence of the null phenotype is higher when disease presents in a more aggressive form (grade IV–V, Breslow $\geqslant 1.5$ mm) (Table II).

No difference in GSTM1 content in positive patients was found between any of the subgroups established.

Immunohistological studies on four of the eight patients a b

**Figure 1** (**a**) GSTM1 showing staining of normal melanocytes in the basal layer, leaving the rest of epidermis practically unstained ($\times$ 330). (**b**) GSTM1 showing moderate staining in MM cells ($\times$ 165).

examined revealed that the antibody to GSTM1 stained the normal melanocytes strongly (in the basal layer), whereas the remaining epidermal cells stained weakly. Cytoplasm and nuclei in melanoma stained moderately (Figure 1). One hundred per cent correlation was found with ELISA blood results.

### Discussion

Excessive UV irradiation, with consequent ROS attack on target molecules such as DNA, appears to be one of the major causes of melanoma. GSTM1 may be important in the repair of DNA since it is present in the normal melanocytes in both cytoplasm and nuclei, and because it may efficiently detoxify peroxides arising in DNA (Ketterer and Meyer, 1989).

Various authors have reported that GSTM1 is almost non-existent in human epidermis (Blacker *et al.*, 1991; Campbell *et al.*, 1991; Raza *et al.*, 1991; Singhal *et al.*, 1993). Minimal amounts of the Mu class GSTs are present in human skin samples, as shown by Western blot analysis or immunohistological staining in the human epidermis, where most cells are keratinocytes. Hence, the risk conferred by the presence or absence of GSTM1 in keratinocytes may also be minimal. However, in melanocytes the opposite is true. Normal melanocytes presented positive GSTM1 staining by immunohistological techniques in the study by Campbell *et al.* (1991), and this was also confirmed here, which suggests that this isoenzyme is important to the host defence of these cells.

GSTM1 deficiency has been studied in relation to lung (Seidegard *et al.*, 1986), bladder and larynx cancer (Lafuente *et al.*, 1993) and in individuals with multiple skin cancers (Heagerty *et al.*, 1994), but this is the first time that this deficiency has been related to melanoma as single tumour.

Our results confirm the importance of phase II enzymes such as GSTM1. Individuals with genetic absence of this isoenzyme appear to be more susceptible to these neoplasms. It also seems that these neoplasms appear in a more aggressive form, since certain negative clinical aspects, such as age at diagnosis, histological types and, especially, the depth of the tumour penetration, tend to depend on the presence of this isoenzyme (Lafuente *et al.*, 1993).

Epidemiological studies suggest that host factors such as fair complexion and a tendency to sunburn rather than tan could be aetiopathogenic factors in malignant melanoma. The distribution of phototypes in MM patients is similar to distribution reported elsewhere (Elwood *et al.*, 1986). However, compared with metabolic risk factors, skin phototypes seem to be independent of GSTM1 phenotype.

Smoking has also been suspected to be related to MM risk (Aubry and McGibbon, 1985); in fact, electrophiles from tobacco smoke could increase the toxicity of ROS, as has been extensively proposed in the literature (Kensler and Trush, 1985; Trush and Kensler, 1991). However, in contrast to what is observed in other tumours (Seidegard *et al.*, 1986; Lafuente *et al.*, 1993), the distribution of GSTM1 phenotypes is similar in smokers and in non-smokers, suggesting that in this case GSTM1 protection is not related to tobacco smoke exposure.

Therefore, it can be concluded that the protective role of GSTM1 operates even in neoplasms that have no direct relationship with tobacco smoke exposure. This provides indirect evidence of the considerable importance of GSTM1 in antioxidant defence, and in the DNA repair system.

**Abbreviations**: DN, dysplastic naevi; GSTM1, glutathione *S*-transferase Mu; MM, malignant melanoma; ROS, reactive oxygen species.

**Acknowledgements**
This work was financed by the Ministry of Health (FIS 93/0028-02). We thank Gabriel Miguel, technician-fellow from CIRIT, for his excellent technical assistance and the Language Advisory Service at the University of Barcelona for correcting the English manuscript.

### References

AUBRY F AND MACGIBBON B. (1985). Risk factors of squamous cell carcinoma of the skin. *Cancer*, **55**, 907–911.

BEITNER H, NORELL SE, RINGBORG U, WENNERSTEN G AND MATTSON B. (1990). Malignant melanoma: aetiological importance of individual pigmentation and sun exposure. *Br. J. Dermatol.*, **122**, 43–51.

BLACKER KL, OLSON E, VESSEY DA AND BOYER TD. (1991). Characterization of glutathione S-transferase in cultured human keratinocytes. *J. Invest. Dermatol.*, **97**, 442–446.

BROCKMÖLLER J, KERB R, DRAKOULIS N, NITZ M AND ROOTS I. (1993). Genotype and phenotype of glutathione S-transferase class $\mu$ isoenzymes $\mu$ and $\varphi$ in lung cancer patients and controls. *Cancer Res.*, **53**, 1004–1011.

CAMBELL JAH, CORRIGALL AV, GUY A AND KIRSCH RE. (1991). Immunohistologic localization of alpha, mu and pi class glutathione S-transferase in human tissues. *Cancer*, **67**, 1608–1613.

ELWOOD JM, WILLIAMSON C AND STAPLETON PJ. (1986). Malignant melanoma in relation to moles, pigmentation, and exposure to fluorescent and other lighting sources. *Br. J. Cancer*, **53**, 65–74.

HEAGERTY AHM, FITZGERALD D, SMITH A, BOWERS B, JONES P, FRYER AA, ZHAO L, ALLDERSEA J AND STRANGE RC. (1994). Glutathione S-transferase GSTM1 phenotypes and protection against cutaneous tumours. *Lancet*, **343**, 266–268.

HUSSEY AJ, STOCKMAN PK, BECKETT GJ AND HAYES JD. (1986). Variations in the glutathione S-Transferase subunits expressed in human livers. *Biochim. Biophys. Acta*, **874**, 1–12.

KENSLER TW AND TRUSH MA. (1985). Oxygen free radicals in chemical carcinogenesis. In *Pathological States*, Vol. III, Oberley LW (eds) pp. 191–236. CRC Press: Boca Raton, FL.

KETTERER B AND MEYER DJ. (1989). Glutathione transferases: a possible role in the detoxication and repair of DNA and lipid hydroperoxides. *Mutat. Res.*, **214**, 33–40.

LAFUENTE A, PUJOL F, CARRETERO P, PEREZ VILLA J AND CUCHI A. (1993). Human glutathione S-transferase $\mu$ (GST $\mu$) deficiency as a marker for the susceptibility to bladder and larynx cancer among smokers. *Cancer Lett.*, **68**, 49–54.

LONGSTRETH JD, LEA CS AND KRIPKE ML. (1992). Ultraviolet radiation and other putative causes of melanoma. In *Cutaneous Melanoma* pp. 46–58. JB Lippincott: Philadelphia.

RAZA H, AWASTHI YC, ZAIM MT, ECKERT RL AND MUKHTAR H. (1991). Glutathione S-transferases in human and rodent skin: multiple forms and species-specific expression. *J. Invest. Dermatol.*, **96**, 463–467.

SEIDEGARD J, PERO RW, MILLER DG AND BEATTIE EJ. (1986). A glutathione transferase in human leukocytes as a marker for the susceptibility to lung cancer. *Carcinogenesis*, **7**, 751–753.

SINGHAL SS, SAXENA M, AWASTHI S, MUKHTAR H, ZAIDI SIA, AHMAD H AND AWASTHI YC. (1993). Glutathione S-transferases of human skin: qualitative and quantitative differences in men and women. *Biochim. Biophys. Acta*, **1163**, 266–272.

TRUSH MA AND KENSLER TW. (1991). An overview of the relationship between oxidative stress and chemical carcinogenesis. *Free Radicals Biol. Med.*, **10**, 201–209.